# Aerobic methane synthesis and dynamics in a river water environment

**DOI:** 10.1002/lno.12383

**Published:** 2023-06-14

**Authors:** Abdullah M. Alowaifeer, Qian Wang, Brian Bothner, Ryan J. Sibert, Samantha B. Joye, Timothy R. McDermott

**Affiliations:** 1 Department of Land Resources and Environmental Sciences, Montana State University, Bozeman, Montana, USA; 2 Department of Microbiology and Cell Biology, Montana State University, Bozeman, Montana, USA; 3 Department of Chemistry and Biochemistry, Montana State University, Bozeman, Montana, USA; 4 Department of Marine Science, University of Georgia, Athens, Georgia, USA

## Abstract

Reports of aerobic biogenic methane (${\text{CH}}_{4}$) have generated new views about ${\text{CH}}_{4}$ sources in nature. We examine this phenomenon in the free-flowing Yellowstone river wherein ${\text{CH}}_{4}$ concentrations were tracked as a function of environmental conditions, phototrophic microorganisms (using chlorophyll a, Chl a, as proxy), as well as targeted methylated amines known to be associated with this process. ${\text{CH}}_{4}$ was positively correlated with temperature and Chl a, although diurnal measurements showed ${\text{CH}}_{4}$ concentrations were greatest during the night and lowest during maximal solar irradiation. ${\text{CH}}_{4}$ efflux from the river surface was greater in quiescent edge waters (71–94 *μ*mol m^−2^ d) than from open flowing current (~ 57 *μ*mol m^−2^ d). Attempts to increase flux by disturbing the benthic environment in the quiescent water directly below (~ 1.0 m deep) or at varying distances (0–5 m) upstream of the flux chamber failed to increase surface flux. Glycine betaine (GB), dimethylamine and methylamine (MMA) were observed throughout the summer-long study, increasing during a period coinciding with a marked decline in Chl a, suggesting a lytic event led to their release; however, this did not correspond to increased ${\text{CH}}_{4}$ concentrations. Spiking river water with GB or MMA yielded significantly greater ${\text{CH}}_{4}$ than nonspiked controls, illustrating the metabolic potential of the river microbiome. In summary, this study provides evidence that: (1) phototrophic microorganisms are involved in ${\text{CH}}_{4}$ synthesis in a river environment; (2) the river microbiome possesses the metabolic potential to convert methylated amines to ${\text{CH}}_{4}$; and (3) river ${\text{CH}}_{4}$ concentrations are dynamic diurnally as well as during the summer active months.

Understanding and reversing global climate change requires that we document the magnitude and underlying (a)biological mechanisms responsible for the generation of the greenhouse gases carbon dioxide (${\text{CO}}_{2}$) and methane (${\text{CH}}_{4}$). ${\text{CO}}_{2}$ concentrations are higher in the atmosphere, but ${\text{CH}}_{4}$ is far more potent in terms of global warming potential (Schaefer 2019). Biologic sources of ${\text{CO}}_{2}$ are varied because it is the primary gaseous product of aerobic respiration. Conversely, biogenic ${\text{CH}}_{4}$ has been viewed traditionally to derive only from anaerobic microorganisms; that is, methanogens that currently are restricted to specific phyla within the *Archaea*. However, evidence of ${\text{CH}}_{4}$ deriving from nonmethanogen biogenic sources, presumably in the presence of oxygen, has been accumulating for over 30 years and has been referred to as the “methane paradox” (reviewed in Tang et al. 2016; Bizic 2021).

The earliest studies of the methane paradox were primarily focused on the marine environment and suggested different methanogen-based mechanisms to explain ${\text{CH}}_{4}$ supersaturation in the presence of oxygen (Oremland 1979; Bianchi et al. 1992; de Angelis and Lee 1994; van der Maarel et al. 1999; Ditchfield et al. 2012). More recent work provided direct evidence that aerobic microbial transformations of methylphosphonate (MPn) can be an important contributor to ${\text{CH}}_{4}$ synthesis in both marine and freshwater environments (Repeta et al. 2016; Yao et al. 2016; Wang et al. 2017). Furthermore, aerobic bacteria can convert methylated amines such as glycine betaine (GB), trimethylamine (TMA), and methylamine (MMA) to ${\text{CH}}_{4}$ (Bižić-Ionescu et al. 2019; Wang et al. 2021). Unexpectedly and perhaps the most extreme paradox is ${\text{CH}}_{4}$ synthesis by phototrophic microbes (Klintzsch et al. 2019; Bižić et al. 2020; Günthel et al. 2020; Bizic 2021, Perez-Coronel and Michael Beman 2022 and see Hilt et al. 2022 for recent review). These observations are supported by lake in situ data documenting ${\text{CH}}_{4}$ production relating to primary production (Bogard et al. 2014) and chlorophyll a (Chl a) levels (León-Palmero et al. 2020; Ordóñez et al. 2023).

The MPn and methylated amine studies discussed above focused on marine and freshwater lake environments. ${\text{CH}}_{4}$ has also been examined in a wide variety of river and stream environments where concentrations are often in excess of saturation (Stanley et al. 2016). As reviewed by Stanley et al. (2016) and in the Global River Methane database (Stanley et al. 2022), ${\text{CH}}_{4}$ concentrations in rivers and streams range from 0 to 456 *μ*mol L^−1^ (Stanley et al. 2022) and are estimated to account for ${\text{CH}}_{4}$ emissions roughly equivalent to that of total coastal and open ocean (Rosentreter et al. 2021). The biological basis that underlies the occurrence of ${\text{CH}}_{4}$ in river systems has not yet been characterized, but at present has been inferred to derive from anaerobic methanogen activity in river benthic environments (Stanley et al. 2016). However, these studies have been strongly biased towards fluvial systems impacted by anthropogenic inputs or activities associated with urban areas and or agriculture (Stanley et al. 2016). Quantifying ${\text{CH}}_{4}$ emissions in riverine systems at any scale is challenging due to a variety of factors that contribute to the complexity of these environments (Stanley et al. 2016; Saunois et al. 2020; Rosentreter et al. 2021; Stanley et al. 2022). Further, work on the topic has varied depending on topography factors, with mountain environments being very poorly represented in the literature (Stanley et al. 2016). A relatively recent study by Flury and Ulseth (2019) sought to examine ${\text{CH}}_{4}$ source(s) in 16 first order oligotrophic mountain streams, concluding that stream water and sediment pore were essentially separate and unlinked compartments with regards to ${\text{CH}}_{4}$ synthesis. They acknowledged oxic pathways for ${\text{CH}}_{4}$ synthesis were possible explanations for carbon isotopic fractionation factors they observed, namely MPn and photodegradation. Subsequent work illustrated that the enzyme reaction that degrades MPn (C-P lyase) would introduce only minimal fractionation (Taenzer et al. 2020) and thus could influence δ^13^C signatures found by Flury and Ulseth (2019).

In a recent study, we documented oxic bacterial ${\text{CH}}_{4}$ synthesis in the thermally stratified Yellowstone Lake, Yellowstone National Park, identifying MMA and GB as substrates that will support this activity (Wang et al. 2021). In contrast to the turbulence and mixing of streams/rivers, distinct zones (depths) in thermally stratified water columns are supersaturated with ${\text{CH}}_{4}$ and lend themselves to the convenient study of the metabolites involved and the metabolic potential of the microbiome at these specific depths (Grossart et al. 2011; Wang et al. 2017, 2021). In the current study, we extended our efforts to assess whether this type of microbial activity occurs in a river environment that is far less structured, but nevertheless should theoretically harbor similar activity. From a combined set of observations, we conclude that aerobic ${\text{CH}}_{4}$ synthesis activity occurs in this river and provide evidence of significant diurnal ${\text{CH}}_{4}$ dynamics.

## Materials and methods

### Study site, sampling, and initial sample analyses

**Fig. 1.**
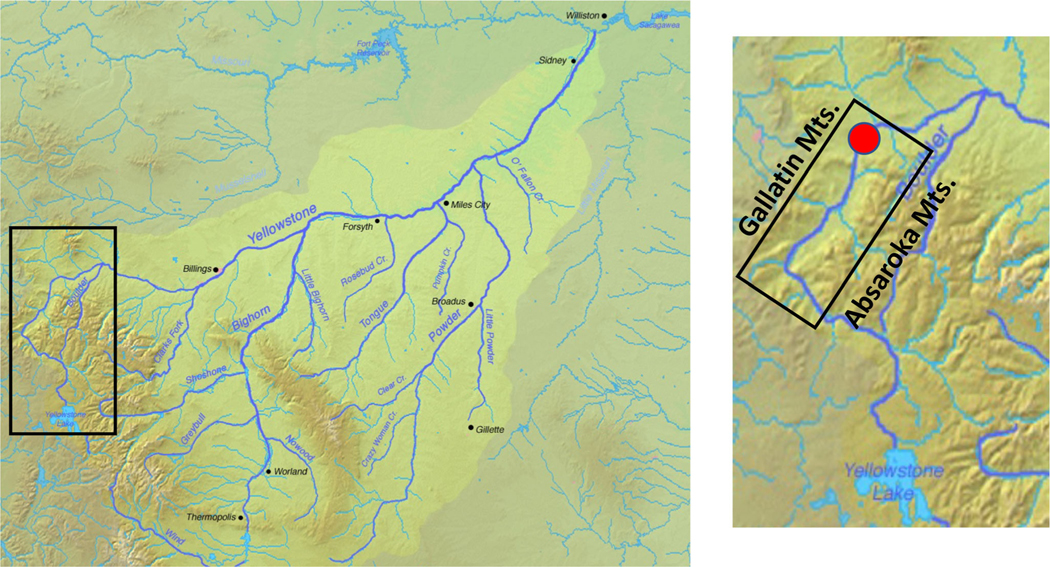
Map of the YR illustrating the drainage area and location of the sampling work. Red dot indicates sampling location. Map created from DEMIS Mapserver (http://www2.demis.nl/mapserver/mapper.asp), which is public domain.

All sampling on the Yellowstone River (YR) occurred ~ 8.2 km south of Livingston MT (Lat., 45.643328/Long., −110.559429) (Fig. 1) where the river is classified as a sixth order stream. During the sampling seasons described in this study, relevant average daily air temperature maxima at the site ranged from 22°C to 30°C during the day and 6°C to 9°C at night (see Supplementary Table S1 for sampling year, date, and approximate time of day samples were taken). Maximum precipitation (primarily as rain) normally occurs in April, May, and June (U.S. Climate data https://www.usclimatedata.com/climate/livingston/montana/united-states/usmt0206), coinciding with maximum snow melt and run off. River hydrographic data (temperature and oxygen) and Chl a were collected using a YSI EXO1 multi-parameter Sonde, and water sampling was as previously described (Wang et al. 2017, 2021). Photosynthetically active radiation (PAR) was measured by lowering a LI-COR LI-193SA spherical underwater quantum sensor to a depth of ~ 15–20 cm beneath the water surface.

For in situ ${\text{CH}}_{4}$ analysis, at each time point river water samples (n=3) were collected by submerging pre-autoclaved 250 mL serum bottles ~ 20 cm beneath the water surface, expelling gas bubbles and then immediately sealing with gray chlorobutyl rubber stoppers that were crimp sealed with an aluminum ring. Samples were then killed immediately by injecting 200 *μ*L of a saturated HgCl_2_ solution and stored on ice for transport to the laboratory where they were stored at 4°C. A 10 mL headspace was introduced (using ultra-high purity ${\text{N}}_{2}$), equilibrated overnight and then ${\text{CH}}_{4}$ analysis of the headspace was conducted using a Varian CP-3800 gas chromatograph with flame ionization detection. ${\text{CH}}_{4}$ concentration in a sample was calculated using Henry’s Law and solubility equations (Wiesenburg and Guinasso Jr 1979). All water samples were then stored in the dark in a cold room (5°C) for subsequent analyses.

### ${\text{CH}}_{4}$ flux measurements

${\text{CH}}_{4}$ flux estimates were obtained using a LICOR LI-7810 ${\text{CH}}_{4}/{\text{CO}}_{2}/{\text{H}}_{2}\text{O}$ Optical Feedback-Cavity Enhanced Absorption Spectrometer (OF-CEAS). The flux chamber was fitted with a foam floatation collar positioned to maintain an internal volume of ~ 2000 cm^3^ with the chamber floating on the water. The analyzer was connected in series to the flux chamber with two 1.5 m lengths of thick-walled, ¼″ Tygon^™^ tubing in a recirculating loop configuration. The atmosphere within the flux chamber was recirculated between the analyzer and chamber using the LI7810 internal diaphragm pump. ${\text{CH}}_{4}$ concentrations within the flux chamber were recorded continuously for ~ 150 s, long enough to establish the linear slopes required to estimate flux.

Measurements were conducted for two different river conditions, in quiescent water locations very near the riverbank (~ 1.0 m deep) and in the flowing river current (~2.5 m deep). For the near riverbank measurements, two separate locations were examined and in each case repeated measurements did not yield different flux estimates and thus are reported as a single measure for each near shore site. Also at each near shore location we physically dislodged the top ~ 10 cm deep sediment/rock material at 0, 1, 3, and 5 m upstream from the flux chamber to experimentally attempt to release ${\text{CH}}_{4}$ as a way to qualitatively assess potential methanogen-based activity at the water-riverbed interface. For the drift flux measurements, the flux chamber was tethered to a raft in a manner whereby the chamber floated freely and without drag. Flux estimates varied between floats and are summarized as the mean and standard deviation of three floats ( ~50 m each) ~10–12 m from the river bank. On that day, the flow rate was ~119 m^3^ s^−1^ (United State Geological Survey monitoring station at Livingston, MT). All measurements had a precision of < 1 ppb (1 standard deviation of repeated blank or standard measurements). A sum of least squares regression fit was applied to the time series data and the slope was used to calculate flux as described by LICOR (https://www.licor.com):

$$F=\frac{\text{dC}}{\text{dt}}*\frac{PV\left(1-w_{0}\right)}{\text{RTS}}$$

where F is ${\text{CH}}_{4}$ flux in units of nmol m^−2^ s^−1^, dC/dt is the change in ${\text{CH}}_{4}$ concentration within the flux chamber per unit time (i.e., ${\text{CH}}_{4}$ velocity) in units of nmol mol^−1^ s^−1^, P is the pressure within the system (in atmospheres), V is the total system volume of the flux chamber, tubing and analyzer (in L), T is the temperature of the system (in Kelvins), R is the ideal gas constant (in L atm K^−1^ mol^−1^), S is the surface area covered by the flux chamber (in m^2^), and w_0_ is the concentration of water vapor in the system. The time-series data generated were approximately linear ($R^{2}$ between 0.95 and 0.99), allowing us to calculate dC/dt as the slope of a least squares regression fit to the data, in units of ppb sec^−1^.

### Analytical methods

Methods and procedures for GB, TMA, dimethylamine (DMA), and MMA analyses were as we previously described in detail (Wang et al. 2021). Briefly, the same bottles collected and HgCl_2_-treated for ${\text{CH}}_{4}$ assays were used for metabolite analysis. ACS reagent grade MMA hydrochloride, DMA hydrochloride, TMA hydrochloride and GB hydrochloride (Sigma Aldrich^®^) were used as standards. For detection and quantification of MMA and DMA, dansyl chloride (Dns-Cl, 1-dimethylaminonaphthalene-5-sulfonyl chloride) was used to label the compounds prior to analysis by liquid chromatography mass spectrometry (LC–MS). Briefly, a Hamilton gastight syringe was used to draw 50 *μ*L water from the sample serum bottle, which was then placed into a 250 *μ*L polypropylene analysis vial and pH adjusted to ~ 9.5 with 2 *μ*L 160 mM sodium hydroxide. Dns-Cl prepared in acetonitrile (20 mg/mL) was added to the sample in a volume of 46 *μ*L. Samples were then incubated for 30 min at room temperature (in the dark). After the incubation period, pH was adjusted to ~ 4 with 2 *μ*L 10% formic acid. The labeled amines were then measured using an Agilent 6538 Q-TOF mass spectrometer equipped with a reverse-phase Agilent Zorbax Eclipse Plus C18 column (2.1 × 150 mm) and operating in positive mode. TMA was assayed as an ethyl bromoacetate derivative. A Hamilton gas tight syringe was used to draw 90 *μ*L water from the sample serum bottle to transfer to a 250 *μ*L polypropylene analysis vial and then 10 *μ*L (20 mg/mL acetonitrile) ethyl bromoacetate was added. Samples were then incubated for 30 min at room temperature. At this point, the sample was ready for analysis. Samples were then analyzed on the same LC–MS system, equipped with a normal-phase Waters ACQUITY BEH HILIC 1.7 mm column (2.1 × 100 mm) and operating in positive mode. GB was assayed without derivatization by mixing 90 *μ*L river water with 10 *μ*L methanol, vortexed for 30 s, and then directly injected into the LC–MS system that was equipped with a normal-phase Waters ACQUITY BEH HILIC 1.7 mm column (2.1 × 100 mm) and operating in positive mode.

### MMA and GB incubations

In the second year of the project, the metabolic capacity of the YR microbiome to convert GB and MMA to ${\text{CH}}_{4}$ was examined. At each sampling, additional separate triplicate 200 mL water samples were collected in sterile bottles and kept on ice during transit to the lab (~ 45 min) where the experiments were immediately set up. A 50 mL subsample for each water sample was transferred to sterile 70 mL serum bottles and aseptically spiked with filter sterilized GB or MMA stock solutions to a final substrate concentration of 1 mM. The serum bottles were plugged with sterile chlorobutyl rubber stoppers, crimped sealed with aluminum rings, and incubated in the dark at room temperature. Headspace ${\text{CH}}_{4}$ was measured immediately (day 0) and then again at a single time point on day 10 using the same GC procedures as described above.

### Statistics

Standard statistical analyses were applied for determining and reporting means and standard errors. To investigate potential relationships between ${\text{CH}}_{4}$ concentrations and environmental and or biological factors, linear or multiple regressions were applied without transformation so as to present the data in its rawest form and to facilitate straightforward visual relationships. Residuals were verified to be normally distributed (Shapiro–Wilk test), and $R^{2}$ and p-values were calculated based on all data points (using least squares analysis). In some cases, regressions are presented by summarizing the data as means and standard error bars to facilitate parameters viewed to be important. For experiments that examined the microbiome potential to convert GB or MMA to ${\text{CH}}_{4}$, data were subjected to ANOVA to determine if differences among means were significant at a p-value ≤ 0.05.

## Results

### Sample site characteristics

**Fig. 2.**
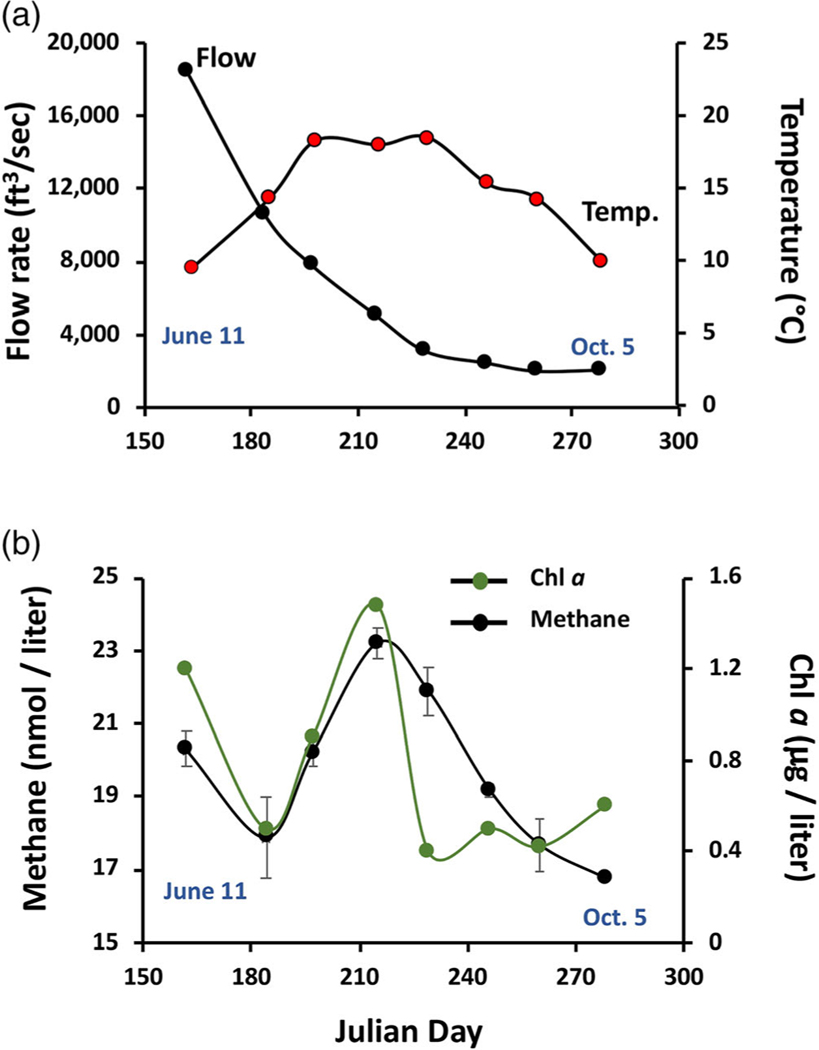
Temporal profiles of river conditions, ${\text{CH}}_{4}$ and phototroph abundance. (**a**) YR temperature and flow rates during the sampling period June 11–October 5, 2020. Flow rate data obtained from the United States Geological Survey flow station USGS 06192500 located near Livingston, MT. (**b**) Chl a (proxy for phototrophs) and ${\text{CH}}_{4}$ during the same sampling dates. Error bars for ${\text{CH}}_{4}$ represent mean ±1 SE, n=4 except for the last sampling date where n=2 due to broken sample bottles.

Sampling was located ~ 190 river kilometers downstream from the outlet of Yellowstone Lake (YR source), which is located in Yellowstone National Park (Fig. 1). The significant distance from the lake outlet and hundreds of contributing lower order tributaries between the lake outlet and the sampling site make the YR sampling environment distinct from our previous study sites within Yellowstone Lake (Wang et al. 2017, 2021). The tributaries upstream of the sampling location drain the east side of the Gallatin and west side of the Absaroka mountain ranges, contributing snow melt derived waters throughout spring and summer months. Initial sampling occurred on June 11, 2020 during high water flows (> 500 m^3^ s^−1^ at the sampling location) (Fig. 2a) and corresponded to a water temperature of 9.5°C (Fig. 2a). Subsequent sampling continued approximately biweekly until October 5, 2020 (Fig. 2a). Throughout the entire sampling season, the EXO SONDE oxygen probe measured dissolved oxygen at 96%–127% of saturation.

### ${\text{CH}}_{4}$ concentrations and flux in relation to environmental factors

The initial ${\text{CH}}_{4}$ concentration of 20.3 nmol L^−1^ decreased on July 3 (Julian days 162–184), but then sharply increased for the following 4 weeks, peaking on August 3 (Julian day 215), after which concentrations steadily declined to the end of the sampling season (Fig. 2b). Overall, ${\text{CH}}_{4}$ concentrations ranged from 17.7 to 23.2 nmol L^−1^, which falls well within the range documented for mountain river environments (reviewed in Stanley et al. 2016). ${\text{CH}}_{4}$ surface flux was measured as a function of water flow and benthic environments. Under near shore quiescent (essentially non-flow) conditions, flux rates for two locations were 94 and 71.0 *μ*mol m^2^ d^−1^ where the benthic environment was comprised of either rocks of various sizes (~ 1–20 cm length) or rocks with some sediments, respectively. Physical disruption of the benthic material at both locations and at various distances from the flux chamber failed to change flux measurements, implying that any ${\text{CH}}_{4}$ release associated with the benthic environment could not be detected by disrupting the uppermost (~10 cm) benthic layer. Flux measured by the floating, non-restrained flux chamber in the rapid current was estimated to be 57 ± 3 *μ*mol m^2^ d^−1^.

**Fig. 3.**
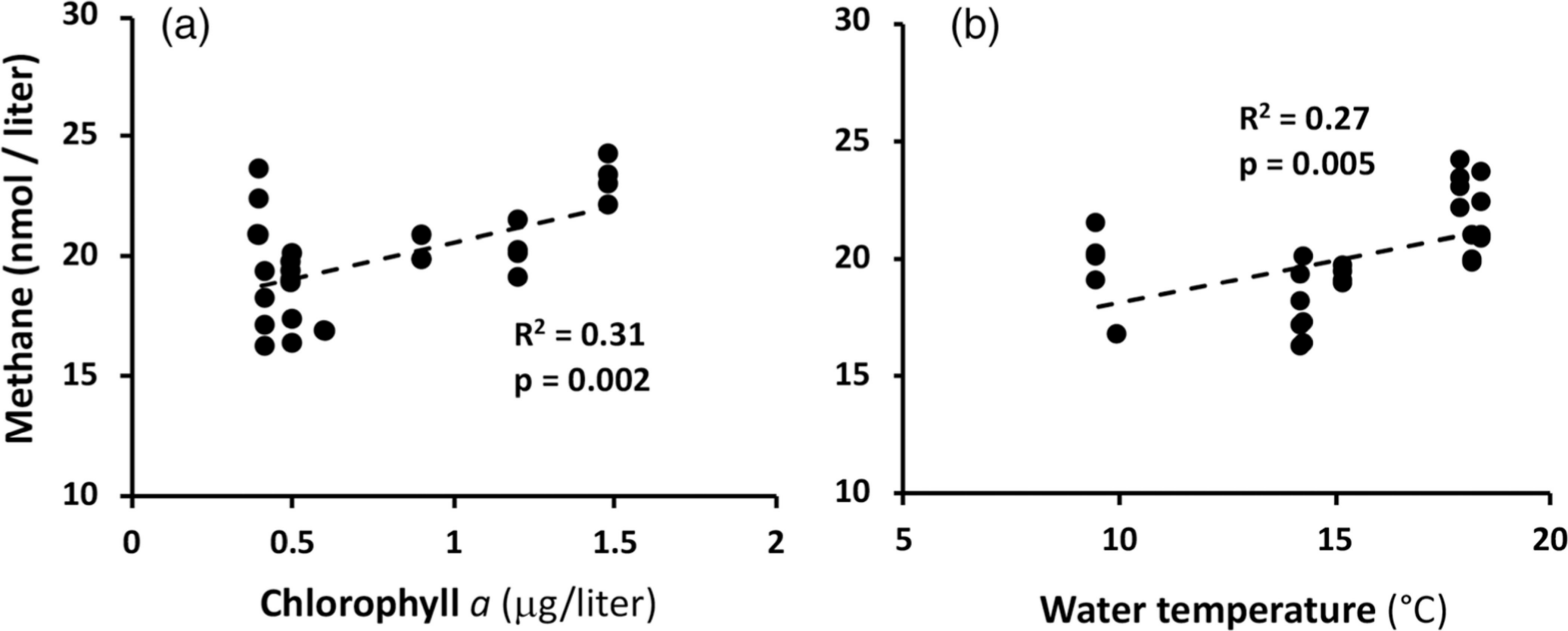
Correlative relationships between YR ${\text{CH}}_{4}$ concentrations and (**a**) chlorophyll a levels and (**b**) water temperature. Samplings included n=4, however some symbols are not visible because of data overlap.

Over the course of the 2020 sampling season, ${\text{CH}}_{4}$ levels largely were superimposable with Chl a, with both peaking during the water temperature plateau lasting from July 16 to August 17 (Fig. 2b). This at least infers the possible involvement of phototrophic microorganisms and is consistent with a prior lake and pond studies (Bogard et al. 2014; Holgerson 2015) and more recent studies providing indirect (Bižić-Ionescu et al. 2019; León-Palmero et al. 2020; Ordóñez et al. 2023) as well as direct evidence (Klintzsch et al. 2019; Bižićet al. 2020; Günthel et al. 2020; Bizic 2021; Perez-Coronel and Michael Beman 2022) of phototrophs (algae and or cyanobacteria) mediating ${\text{CH}}_{4}$ synthesis. Regression analysis suggests a significant correlation between ${\text{CH}}_{4}$ and Chl a ($R^{2}$, p-value = 0.002; Fig. 3a), and that both parameters track with water temperature as expected for a biogenic source ($R^{2}=0.27$, p-value = 0.005, Figs. 2, 3b). A multiple regression model including both temperature and Chl a illustrated that these parameters were additive in terms of predictive power, yielding an adjusted $R^{2}=0.57$ and p-value < 0.00001.

### Diurnal studies

**Fig. 4.**
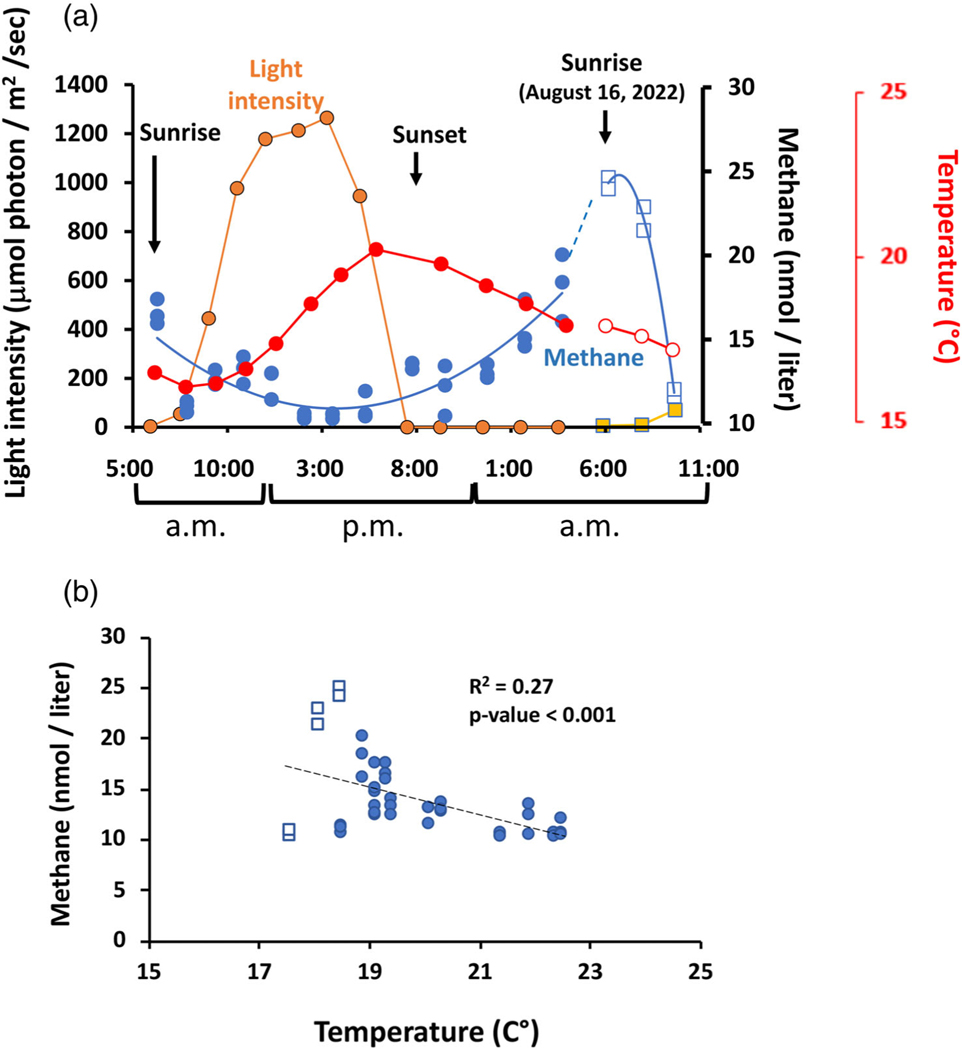
Diurnal ${\text{CH}}_{4}$ cycling on the YR. (**a**) ${\text{CH}}_{4}$ concentration, water temperature and solar radiation intensity profiles as a function of time of day. (**b**) Regression analysis of ${\text{CH}}_{4}$ concentration vs. water temperature during the diurnal sampling periods. $R^{2}$ value represents all data points from both sampling dates. Square symbols represent data collected 11 d following collection of the data shown as circles. Dashed line in panel A represents a hypothetical trend line connecting the two different data sets, whereas the dashed line in panel B represents the least squares linear regression estimate of all data.

Given the evidence of phototrophic ${\text{CH}}_{4}$ production in this and other studies, additional work was conducted in 2022 to assess ${\text{CH}}_{4}$ concentrations as a function of PAR at roughly the same depth from which water samples were taken. The initial approach involved a diurnal sampling scheme, which commenced at sunrise (5:55 h local time) with subsequent sampling at roughly 1.5–2.0 h intervals over the course of ~ 22 h (Fig. 4a). As PAR increased, ${\text{CH}}_{4}$ concentrations decreased from an apparent overnight maximum and decreased further during maximum PAR. As PAR levels went to essentially zero at sunset, ${\text{CH}}_{4}$ increased continually through overnight monitoring. Diurnal ${\text{CH}}_{4}$ dynamics of this nature were not anticipated, particularly the observed decrease associated with sunrise. Therefore, a follow-up sampling was conducted (11 d subsequent; open symbols in Fig. 4a,b). While ${\text{CH}}_{4}$ concentrations at sunrise differed between the two sampling dates, ${\text{CH}}_{4}$ was again observed to decrease during sunrise and the early morning hours.

Water temperature ranged from 17.7°C to 22.5°C during the continuous sampling period. As expected, the temperature maximum was phase shifted relative to maximum PAR because of the time required for the water to absorb solar energy and warm. Interestingly, the temperature profile was nearly a mirror image reflection of the ${\text{CH}}_{4}$ profile (Fig. 4a). This suggests short term water temperature and ${\text{CH}}_{4}$ concentration were inversely related, which is illustrated in Fig. 4b. Linear regressions between ${\text{CH}}_{4}$ and water temperature yielded an $R^{2}$ of 0.27 across both sampling days, but 0.33 for continuous data acquired only on the first day. Both regressions were judged to be significant based on their p-values < 0.0001.

### Methylated amines

**Fig. 5.**
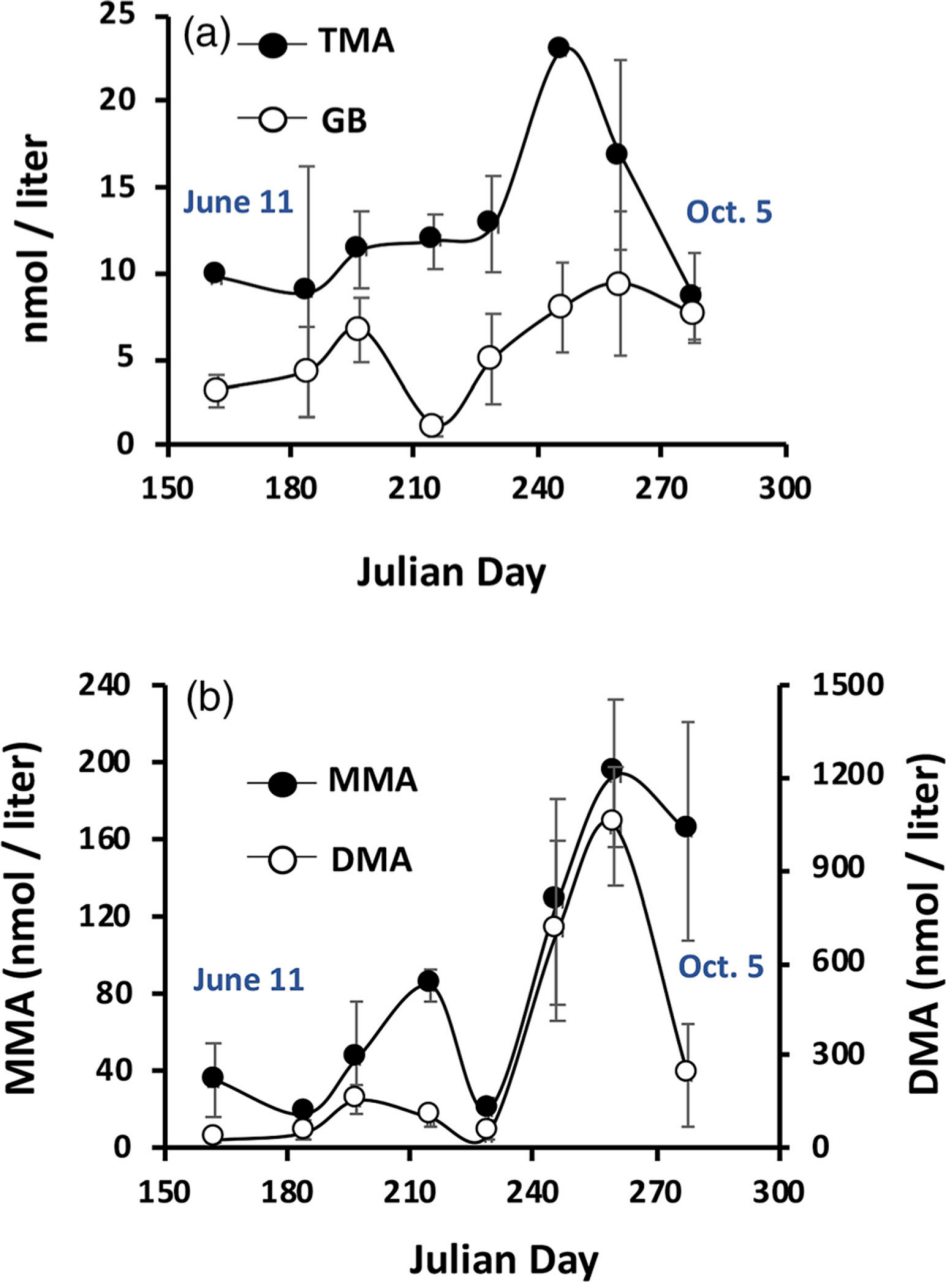
Temporal profiles of GB, TMA, DMA, and MMA in YR water samples taken near Livingston MT in 2020. Symbols and error bars represent mean ± 1 SE, n=4 except for the last sampling date where n=2 due to broken sample bottles.

Our prior efforts on Yellowstone Lake and with Yellowstone Lake bacterial isolates (Wang et al. 2021) and those of the Grossart group on Lake Stechlin (Bižić-Ionescu et al. 2019), documented direct evidence that methylated amines can support or enhance aerobic biogenic ${\text{CH}}_{4}$ production. As such, we examined the water samples for GB, TMA, DMA, and MMA. When detected, GB levels ranged from 1.1 to 23.9 nM, and TMA ranged between 3.7 and 24.6 nM (Fig. 5a). DMA and MMA levels were substantially higher, ranging from 4.2 nM to ~ 1.5 *μ*M and from 4.7 to 280 nM, respectively (Fig. 5b). We note with interest the sharp (but highly variable) increase in all four methylated metabolites that coincided with cooling water temperatures and the steep decrease in Chl a levels (compare Figs. 2b, 5).

**Fig. 6.**
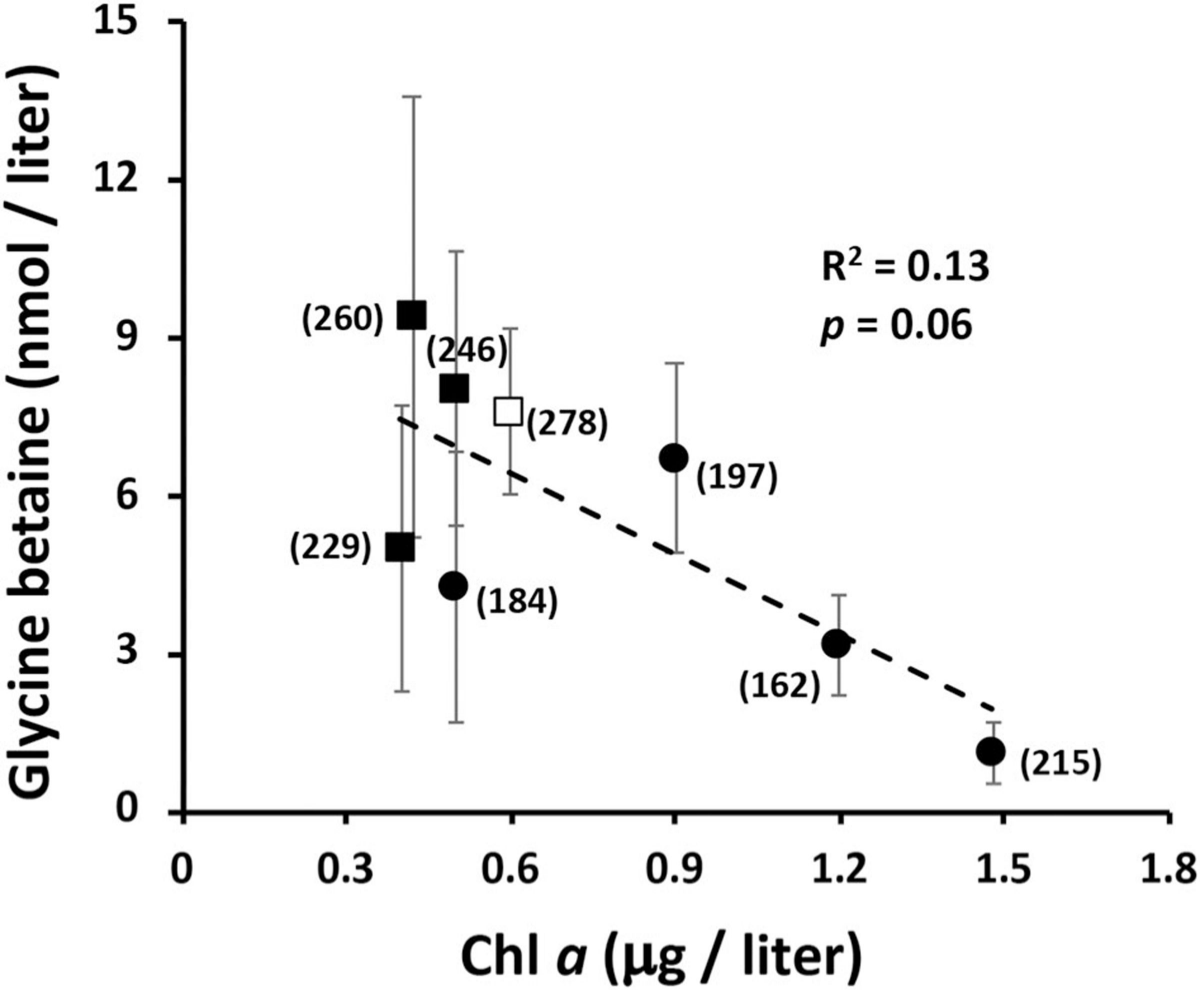
Regression analysis assessing the relationship between the phototroph proxy marker Chl a and GB concentrations. Square symbols denote data from the last four sampling points coinciding with Chl a decline. All symbols ± error bars illustrate the GB means and standard error for each sampling time (n=4) except for the open symbol where n=2 (±range) due to broken sample bottles. Each data symbol are labeled with respect to the Julian day represented. $R^{2}$ and p-value summarize statistical analysis of all data points summarized by the means ± SE.

**Fig. 7.**
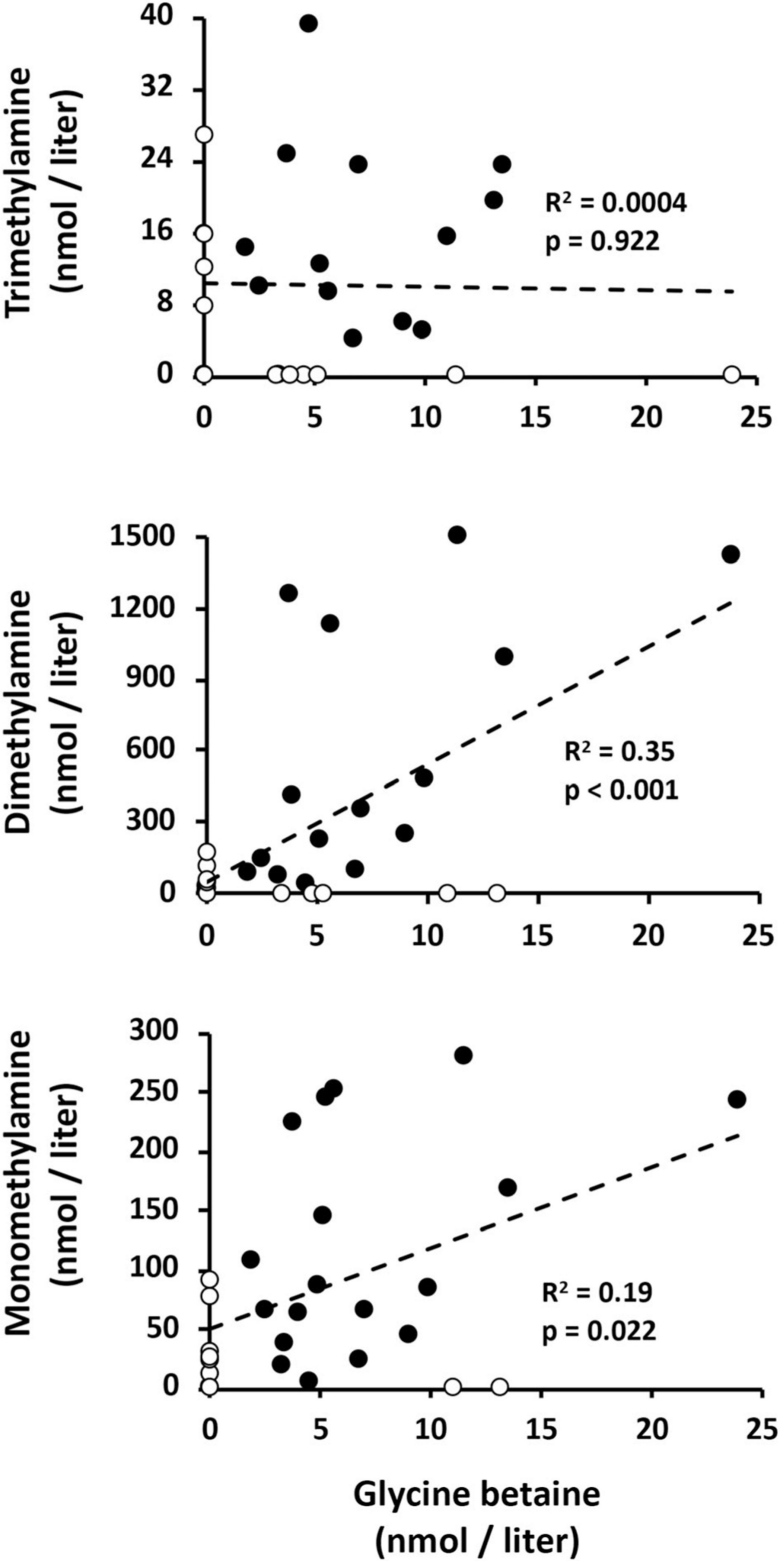
Regression analysis of untransformed data relating GB with TMA, DMA, and MMA. Some data points are not visible because they are hidden beneath other data points. Empty symbols indicate the metabolite was below the detection limit but assigned as zero. $R^{2}$ and p-value summarize statistical analysis of all data points, untransformed.

**Fig. 8.**
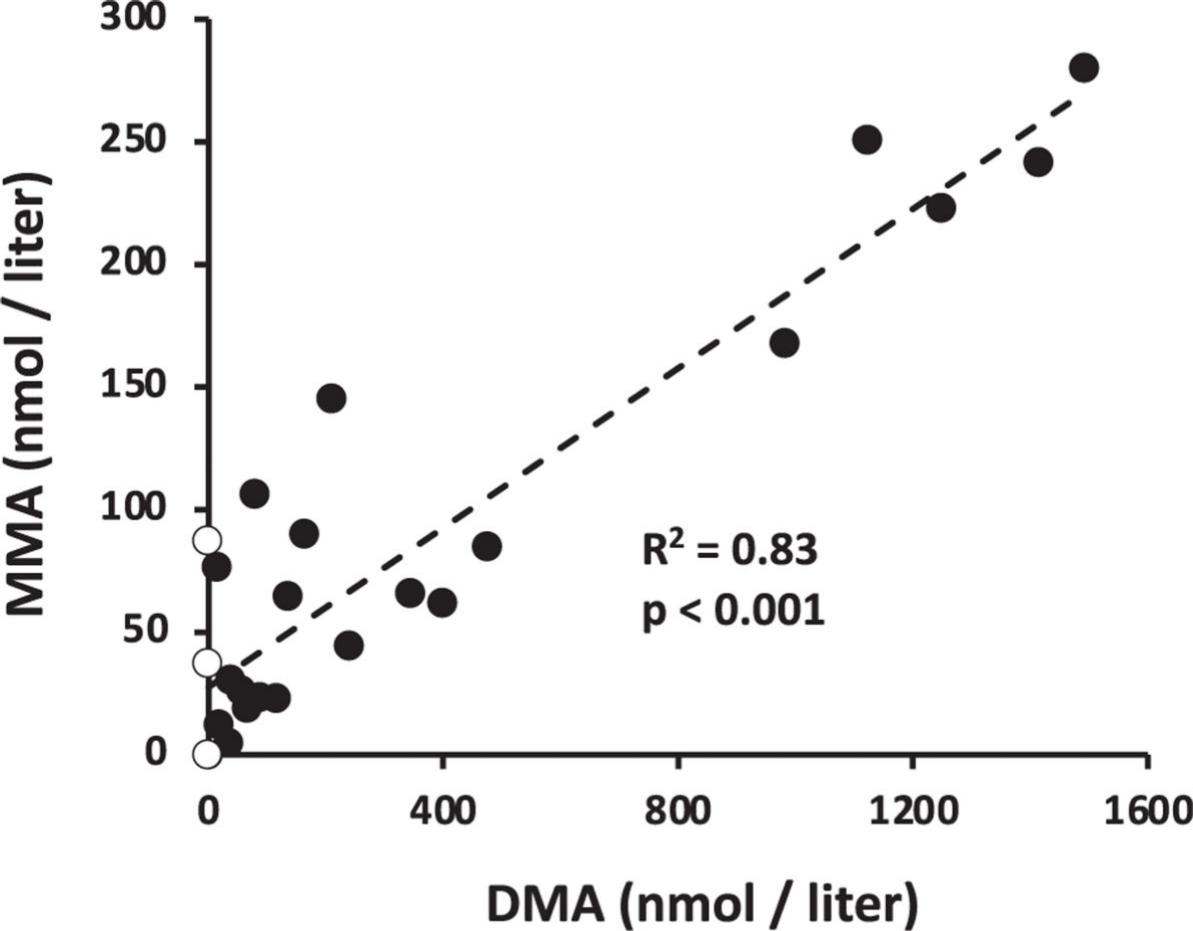
Regression analysis examining the relationship between DMA and MMA over the course of the 2021 sampling season. Empty symbols indicate the metabolite was below the detection limit but assigned as zero. $R^{2}$ and p-value summarize statistical analysis of all data points, untransformed.

We interpret the Chl a decline as marking some type of lytic event for one or more abundant phytoplankton species, suggesting the possibility of cell lysis resulting in the release of these compounds. GB has previously been associated with phototrophic mats (King 1988; Oren 1990), and thus it might be reasonable to expect it to be a metabolite released by such an event. Regression analysis of these metabolites were consistent with this view; that is, maximal GB was recorded at the lowest Chl a levels (Fig. 6). GB concentrations did not correlate with TMA, but did with DMA ($R^{2}=0.35$, p<0.001) and weakly with MMA ($R^{2}=0.19$, p<0.022; Fig. 7). And as would be predicted from Fig. 5b, DMA and MMA were strongly correlated ($R^{2}=0.83$, p<0.001) (Fig. 8). These co-varying relationships are consistent with a degradation pathway of GB→DMA→MMA.

**Fig. 9.**
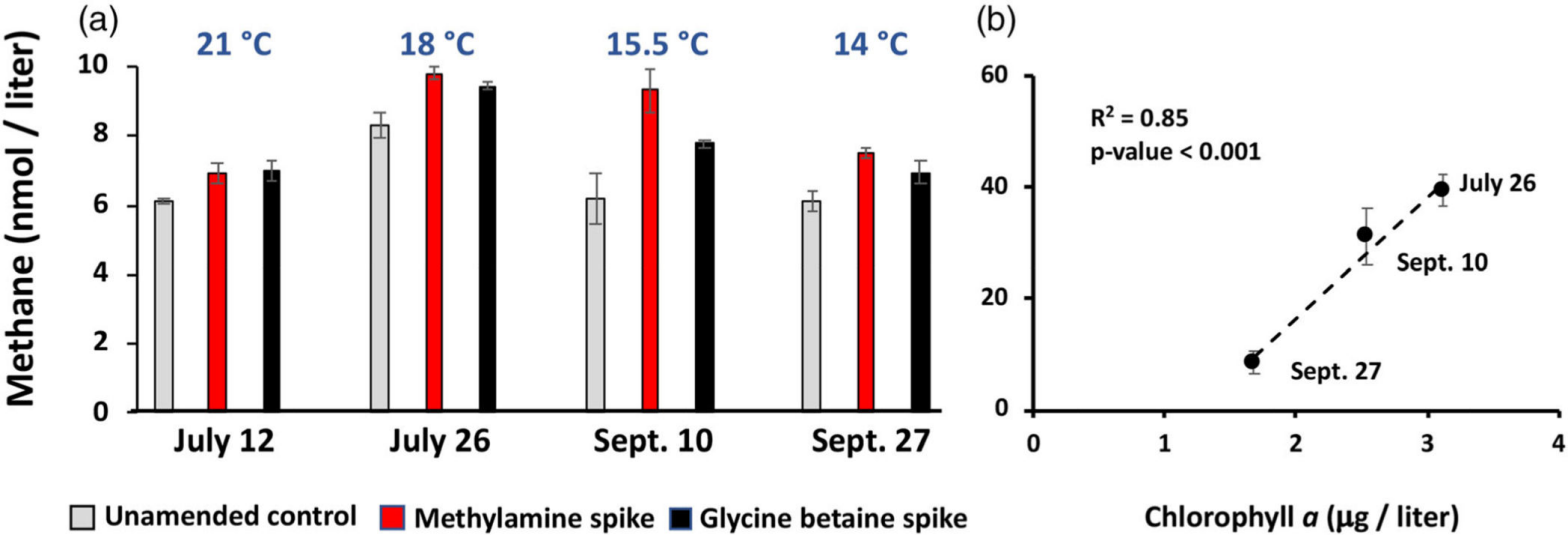
Aerobic ${\text{CH}}_{4}$ production potential from GB or MMA in YR water samples taken at the dates shown in 2021. (**a**) Samples (n=3) were either unamended (control) or spiked with MMA or GB. Note that ${\text{CH}}_{4}$ concentrations in control bottles did not change from the day the water was sampled (t=0). Data and error bars represent mean ± SE; all spike treatments were statistically different from respective unamended controls, p-values ranged from < 0.001 to 0.027. (**b**) Regression analysis illustrating the relationship between Chl a and in situ ${\text{CH}}_{4}$. Note: the sonde instrument was under repair and not available for measuring Chl a for the July 12 sampling.

It was of interest to determine if any of these metabolites could be associated to ${\text{CH}}_{4}$ synthesis, as previously reported for lake environments (Klintzsch et al. 2019; Bižić et al. 2020; Günthel et al. 2020; Wang et al. 2021). To more directly address this question, near-surface water samples were collected in 2021 and incubated either unamended or spiked with GB or MMA. After 10 d incubation period, all samples spiked with GB or MMA presented significantly more ${\text{CH}}_{4}$ than the unamended controls (Fig. 9a), in some cases exceeding controls by as much as two-fold (MMA, Sept. 10). As with the prior sampling season (Fig. 3a), river ${\text{CH}}_{4}$ concentrations in these samples illustrated the same, though much stronger, positive relationship between ${\text{CH}}_{4}$ and Chl a ($R^{2}=0.85$) and temperature ($R^{2}=0.73$; Fig. 9b). Further, the same seasonal pattern was observed; that is, Chl a, temperature, and ${\text{CH}}_{4}$ levels decreased as sampling progressed into the cooler autumn months (Fig. 9b). Collectively, two different years of seasonal sampling (Figs. 3a, 9b) illustrated the same positive relationships between Chl a, temperature and ${\text{CH}}_{4}$.

## Discussion

The enigma referred to as the “methane paradox” has been scrutinized for decades, with significant progress being made towards understanding its biological basis. Clearly, MPn (Karl et al. 2008; Valle and Karl 2014; Repeta et al. 2016; Yao et al. 2016; Wang et al. 2017) and methylated amines (Bižić-Ionescu et al. 2019; Wang et al. 2021) can be used as substrates to support aerobic ${\text{CH}}_{4}$ synthesis. However, there remains much work to determine the extent to which this occurs and relative importance across different environments. Work conducted herein expands beyond lake work that dominates current freshwater literature concerning aerobic ${\text{CH}}_{4}$ synthesis. Lake water columns offer the convenience of locating ${\text{CH}}_{4}$ maxima at defined depths, which enables focused efforts such as isotope labeling, culturing, and omics-based approaches to characterize relevant organisms and metabolites (Grossart et al. 2011; Günthel et al. 2019; Wang et al. 2021), as well as probing for biogeochemical relationships that can be linked to sort out organisms, functions and fates (Yao et al. 2016; Bižić-Ionescu et al. 2018; Wang et al. 2021). By contrast, river environments can be conceptualized as water flowing over a substratum of assorted composition with potential functional linkages between the river benthos and surface waters significantly constrained by water depth, flow rates, and turbulence.

The single time point ${\text{CH}}_{4}$ flux measurements conducted herein found near shore quiescent waters to be greater than that registered for flowing current. All of these measurements suggest the YR could potentially yield more ${\text{CH}}_{4}$ than other mountain rivers thus far examined (reviewed by Stanley et al. 2016), although clearly this awaits an expanded diurnal and seasonal assessment. The current study clearly does not rule out anaerobic methanogenesis in the river benthos as a contributor to the observed ${\text{CH}}_{4}$ and indeed it would be expected to respond to increased temperature, as was documented here (Fig. 3b). However, given the nature of the YR bed (meters deep rocks) and strength of the river current, it is not physically possible to secure a chamber device to the river bottom in order to assess potential benthic contributions to river ${\text{CH}}_{4}$. The relative contribution of benthic biogeochemistry to water column ${\text{CH}}_{4}$ could increase as river volume decreases (Gardner and Martin 2018) but this was not the case during this seasonal sampling campaign (Fig. 2). Further, an analysis of major North American rivers has led to the suggestion that water column processes increasingly dominate in rivers greater than fifth order (Gardner and Martin 2018), which includes the study location on the YR.

In the quiet water near the bank, attempts to induce an artificial ebullition type event by physically disrupting the water-benthic interface (top 0–10 cm at ~ 0.5 m water depth) at various distances from the floating flux chamber failed to generate anomalous ${\text{CH}}_{4}$ readings or a change in ${\text{CH}}_{4}$ flux. However, this is not necessarily determinative because the source(s) of anaerobic methanogen-based ${\text{CH}}_{4}$ in this particular river could very well derive from deeper locations in the river bed profile. This would be exceedingly difficult to quantify in the YR and other rivers like it. Lateral flow could be a contributor to the increased ${\text{CH}}_{4}$ flux of these near shore sites (Leith et al. 2014; Rasilo et al. 2017; Leng et al. 2022), which were conducted 3–5 m from the river bank composed of muds and sandy sediments. Lateral flow is a phenomenon argued to occur across even much longer distances in freshwater lakes (Fernández et al. 2016; DelSontro et al. 2018).

In lower elevation environments, river flow rates slow and temperatures rise, in contrast to higher elevation mountain rivers such as the YR, which has strong currents, lower seasonal temperatures, and is oxygen saturated. In such environments it is difficult to define linkages between specific organisms, metabolites, and end products. Regression analyses do not prove cause and effect, but nevertheless are useful for revealing potential organism-metabolite relationships. Regression analysis conducted in the current study sought to determine if relationships demonstrated in prior studies might also be revealed in the YR. Specifically, phototrophic organisms are directly or indirectly involved in ${\text{CH}}_{4}$ synthesis (Grossart et al. 2011; Bižić et al. 2020, León-Palmero et al. 2020; Bizic 2021). In this study, we used Chl a as a proxy for tracking phototrophs and relating phototrophs with ${\text{CH}}_{4}$ concentrations (Figs. 2a, 9b), observing similar positive correlations as previously documented in lake studies (León-Palmero et al. 2020; Ordóñez et al. 2023). GB occurrence has been linked to phototrophs (King 1988) and the weak correlation observed here suggests GB could be a ${\text{CH}}_{4}$ precursor (Fig. 6), although the relationship is limited by the significant variability in the GB data in the last four samplings that is reasonably assigned to an apparent phototroph lytic event (Figs. 2, 5).

We are unaware of any previous studies documenting GB or TMA in river waters, and thus the current study provides novel information in this regard. The MMA and DMA levels observed in the YR are consistent with that described previously in seven other rivers, reporting as high as 663 and 264 nM (reviewed in Poste et al. 2014). However, while prior studies have linked these methylated amines to aerobic ${\text{CH}}_{4}$ synthesis (Bižíc-Ionescu et al. 2019; Klintzsch et al. 2019; Bižíc et al. 2020; Günthel et al. 2020; Wang et al. 2021), the variable surge in these metabolites (Fig. 5) occurred while river ${\text{CH}}_{4}$ levels were decreasing (Fig. 2), implying these metabolites were consumed for purposes other than ${\text{CH}}_{4}$ synthesis. Because methylated amines are considered to be indicator metabolites of GB degradation (King 1988; Lee 1988; Oren 1990; Müller et al. 2009), the simultaneous increase of TMA, DMA, and MMA (Fig. 5) is likely not coincidental. Correlations among these metabolites (Fig. 7) is consistent with a degradation pathway of GB→DMA→MMA, with the caveat being that GB stability would appear to be short lived, being turned over rapidly to TMA and or DMA, and that GB degradation may proceed through mechanisms other than the above pathway.

Overall, DMA levels across the sampling period were roughly six-fold greater than MMA (Fig. 8). This is perhaps explained by DMA having environmental fates other than MMA production and would also at least partially account for the weaker relationship between GB and MMA; that is, each intermediate in a degradation pathway can be diverted such that correlative relationships with the initial substrate decreases at each step. MMA is well documented as a carbon source for methylotrophs (Chistoserdova et al. 2009; Chistoserdova 2011), used as a nitrogen source by non-methylotrophs via the MMA oxidative pathway (Latypova et al. 2010; Taubert et al. 2017) or as an energy source for methylovores (Sun et al. 2011). Consequently, there are several competing uses and fates for MMA that, in this particular environment and sampling period, could be viewed to exceed its conversion to ${\text{CH}}_{4}$. Nevertheless, both GB and MMA can support aerobic ${\text{CH}}_{4}$ synthesis (Wang et al. 2021) as was shown in the current study of a river microbiome (Fig. 9). As such, we can conclude that the metabolic potential for this conversion exists and warrants additional study.

One of the most interesting aspects of this study is the pattern of diurnal ${\text{CH}}_{4}$ cycling (Fig. 4a), which we anticipate will have both biological and physicochemical explanations. There are similarities between what was observed in these diurnal ${\text{CH}}_{4}$ measurements and those reported by Bižić et al. (2020) in their work with cultures of *Microcystis aeruginosa* and *Prochlorococcus* sp. MIT0604. Specifically, phototroph cultures showed a delayed increase in ${\text{CH}}_{4}$ concentration relative to maximum photosynthesis (as inferred by O_2_ generation), peak experimental photoperiod, and light intensity, which is very similar to what was observed in this study (Fig. 4a). ${\text{CH}}_{4}$ decreased during the dark period of their experiment due to off-gassing, which no doubt occurs in open environments such as the YR. However, the minimum ${\text{CH}}_{4}$ concentrations during maximum solar irradiance observed in the current study are difficult to reconcile with that pure culture work (Bižić et al. 2020) or lake water incubations (Perez-Coronel and Michael Beman 2022), where ${\text{CH}}_{4}$ synthesis positively correlated with photosynthesis and increasing light level [17].

In this study, the ${\text{CH}}_{4}$ minimum occurred during peak irradiance (~ 1250 *μ*mol m^−2^ s^−1^, Fig. 4), which was two- to ten-fold higher than that used in either of these aforementioned studies. This suggests the possibility of photoinhibition, which is a well-documented phenomenon for phototrophs (Han et al. 2000; Raven 2011; Zavafer and Mancilla 2021) that adversely affects all phytoplankton (Litchman and Klausmeier 2008), and can occur well within the time frame observed here (Lima-Melo et al. 2019). In lake environments, phytoplankton can acclimate to deeper regions of the water column, but a turbulent river environment prevents seeking such an escape. Microbial biofilms in the benthic environment could provide some protection from ultraviolet radiation (Elasri and Miller 1999; Bernbom et al. 2011; Kviatkovski et al. 2018), although the relative importance of UV or photoinhibition refuge would depend on water depth and seasonal-dependent sediment loads carried by the river (e.g., spring snow melt or rainfall induced erosion), both of which would attenuate irradiance penetration to benthic biofilms.

We draw attention to the different relational patterns observed for ${\text{CH}}_{4}$ concentrations and water temperature. Across two seasonal studies we documented a positive relationship between ${\text{CH}}_{4}$ concentration and increasing water temperature (Figs. 3b, 9b), which is expected based on basic biological responses to increased temperature (methanogen as well as non-methanogen). By contrast, in the short term diurnal studies aqueous ${\text{CH}}_{4}$ concentrations were negatively correlated with temperature; an increase of ~ 5°C during the day corresponded to an almost two-fold decrease in ${\text{CH}}_{4}$ concentrations (Fig. 4a). Methanotrophs are components of a river microbiome (Erwin et al. 2005; Burrows et al. 2021; Bussmann et al. 2021) and thus it could perhaps be argued that their activity could increase with temperature, potentially exceeding ${\text{CH}}_{4}$ synthesis, regardless of its source. However, this putative methanotroph activity occurred during peak irradiance (Fig. 4a), which conflicts with other studies that have found light can inhibit ${\text{CH}}_{4}$ oxidation (Dumestre et al. 1999; Murase and Sugimoto 2005; Thottathil et al. 2019).

In a turbulent river, mixing is significant and thus water temperatures should be relatively uniform with depth. If benthos-related methanogens were important to the observed ${\text{CH}}_{4}$ concentrations, they should have readily responded to increased temperature. This was not the case and is consistent with our failed attempts to illustrate ${\text{CH}}_{4}$ release by physically disrupting the rocks in the YR bed. If ${\text{CH}}_{4}$ derived primarily from methanogens deep in the sub-surface benthos profile, temperature change at depth would be expected to be temporally phase shifted relative to the daytime maximum, peaking hours after the water temperature maximum and thus merely coincided with conditions where photosynthesis was no longer possible. However, if the increased night-time ${\text{CH}}_{4}$ concentrations were directly tied to the dominant phytoplankton occurring in the YR during this portion of the study, then this at least suggests that ${\text{CH}}_{4}$ synthesis can derive from stored carbohydrate generated during the window of time where photosynthesis was more efficient before or following the high photon flux intensity.

Finally, it is at least tenable to consider that decreased ${\text{CH}}_{4}$ during the day period is not due to decreased synthesis or increased consumption activity, but rather derives from temperature influenced gas behavior. Gas exchange velocity increases significantly and rapidly in response to temperature increase (Hall Jr. and Ulseth 2020), which in turn translates to increased flux. Rovelli et al. (2018) concluded that river water temperature accounted for up to 46% of the variability in the O_2_ gas transfer velocity on an hourly scale. Further, in a diurnal lake study (Sieczko et al. 2020), maximum and minimum ${\text{CH}}_{4}$ flux corresponded to day and night periods, respectively, and corresponded to maximal temperature change. Simply put, in the diurnal experiments if ${\text{CH}}_{4}$ flux rate exceeds ${\text{CH}}_{4}$ synthesis rate, then ${\text{CH}}_{4}$ concentrations would be expected to decrease. Potentially at least, short-term temperature effects could significantly influence ${\text{CH}}_{4}$ modeling efforts in any environment. Incorporating diurnal temporal dynamics of ${\text{CH}}_{4}$ levels and flux as a function of water temperature in river and stream environments would likely strengthen our understanding of ${\text{CH}}_{4}$ synthesis and fate, enhancing modeling efforts at all scales.

When viewed collectively, the data compiled in this study illustrate the metabolic potential of the YR microbiome to aerobically generate ${\text{CH}}_{4}$. Determining the specific role of the apparent relevant phototroph(s) will require significant effort. We conclude that the YR phototroph(s) were either directly generating ${\text{CH}}_{4}$ or feeding physically associated bacteria, perhaps in a syntrophic manner wherein viability of all members is prerequisite for ${\text{CH}}_{4}$ synthesis. It is not unreasonable to suggest that the observed increases in GB, DMA, and MMA derived from a phototroph lytic event. However, the temporal increase of these metabolites did not result in a ${\text{CH}}_{4}$ surge, which could be due to increased YR microbiome consumption by a wide range of bacteria inhabiting river environs (Boden et al. 2008) via pathways not leading to ${\text{CH}}_{4}$, or a surge in methanotrophs responding to this release. Still and regardless, direct addition of GB and MMA to YR water samples resulted in significantly increased ${\text{CH}}_{4}$ relative to controls (Fig. 9a). The increased ${\text{CH}}_{4}$ in the spiked samples (Fig. 9a) does not quantify synthesis rates nor accounts for potential methanotroph or methylotroph activities that would constrain the apparent conversion of GB or MMA to ${\text{CH}}_{4}$. However, these experiments do provide direct evidence that the YR microbiome at least has the metabolic potential to convert these metabolites to ${\text{CH}}_{4}$ under oxic conditions. The exact mechanism is unknown and indeed the pathway could conceivably be convoluted, but the lack of light in the incubations would seem to argue that the effect of these methylated amines was not to boost photosynthesis per se as a prelude to ${\text{CH}}_{4}$ synthesis.

## Supplementary Material

SUPINFO1

## Data Availability

The data that support the findings of this study are available in this article and Supporting Information are available from the corresponding author upon reasonable request.
