# Supplementary material for: Aerobic methane synthesis and dynamics in a river water environment
Source: Limnol Oceanogr. Author manuscript; Available in PMC 2023 Nov 24. (PMC10624334; doi:10.1002/lno.12383)

# Supplementary Information for manuscript:

**Aerobic Methane Synthesis and Dynamics in a River Water Environment**

**By**

**Abdullah M. Alowaifeer, Qian Wang, Brian Bothner, Ryan J. Sibert, Samantha B. Joye, Timothy R. McDermott**

Contains: 1 table


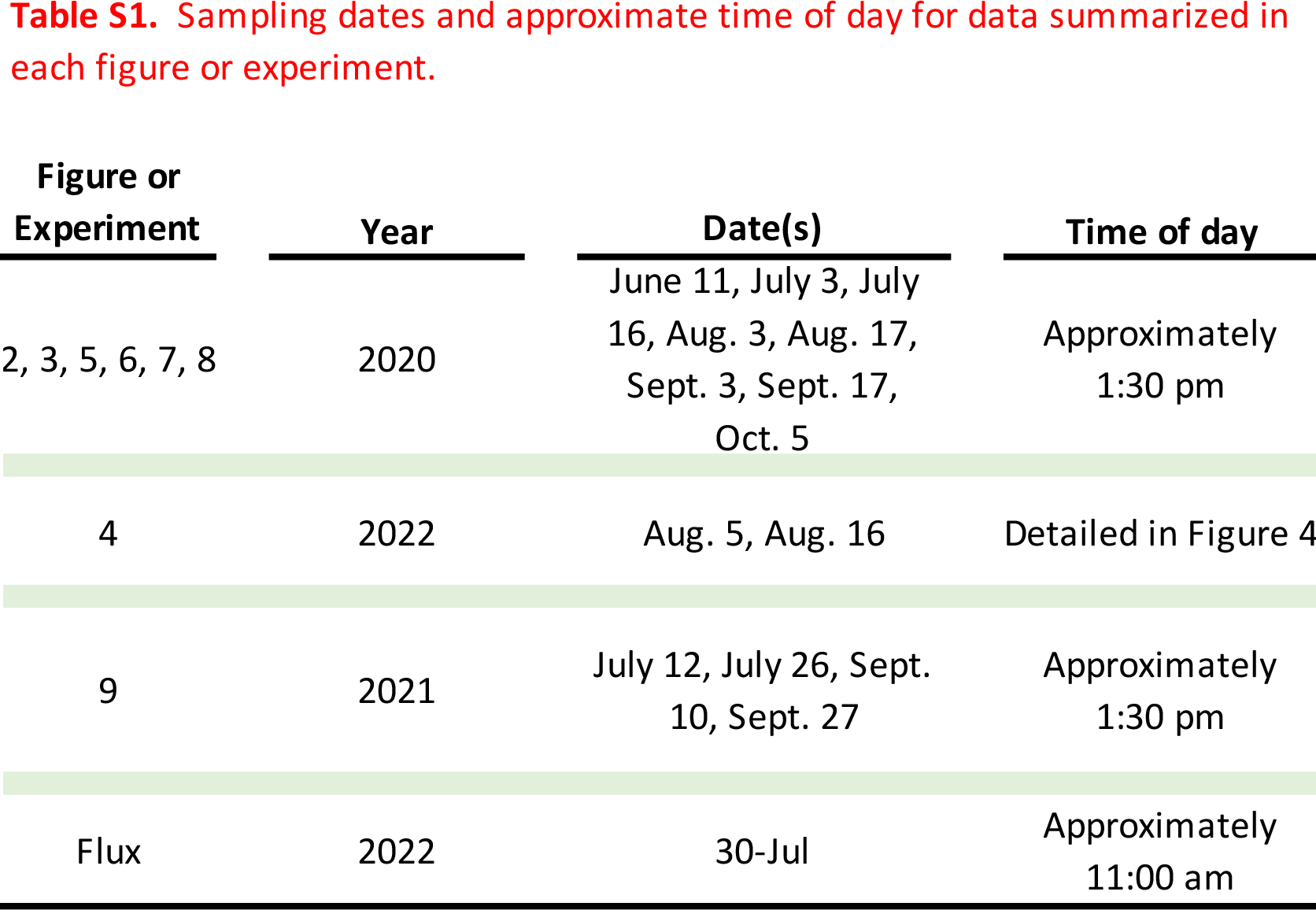

Supplement: SUPINFO1 [file NIHMS1904831-supplement-SUPINFO1.docx]
